# Crystal structure of 3-(3,4,5-tri­meth­oxy­phen­yl)-1,2,3,4-tetra­hydro­cyclo­penta[*b*]indole-2-carb­oxy­lic acid

**DOI:** 10.1107/S2056989015008786

**Published:** 2015-05-13

**Authors:** Daniara Fernandes, Deborah de Alencar Simoni, Manoel T. Rodrigues, Marilia S. Santos, Fernando Coelho

**Affiliations:** a Laboratory of Synthesis of Natural Products and Drugs, Institute of Chemistry, University of Campinas, PO Box 6154 – 13083-970, Campinas, SP, Brazil; b Laboratory of Single Crystal X-Ray Diffraction, Institute of Chemistry, University of Campinas, PO Box 6154 – 13083-970, Campinas, SP, Brazil

**Keywords:** crystal strycture, indole skeleton, Morita–Baylis–Hillman adduct, hydrogen bonding

## Abstract

In the title compound, C_21_H_21_NO_5_, obtained from a Morita–Baylis–Hillman adduct, the hydrogenated five-membered ring adopts a shallow envelope conformation, with the C atom bearing the carb­oxy­lic acid substituent deviating by 0.237 (1) Å from the mean plane of the other four atoms (r.m.s. deviation = 0.007 Å). The dihedral angle between the fused ring system (all atoms; r.m.s. deviation = 0.057 Å) and the pendant trimeth­oxy benzene ring is 66.65 (3)°. The C atoms of the *meta*-meth­oxy groups lie close to the plane of the benzene ring [deviations = 0.052 (1) and −0.083 (1) Å], whereas the C atom of the *para*-meth­oxy group is significantly displaced [deviation = −1.289 (1) Å]. In the crystal, carb­oxy­lic acid inversion dimers generate *R*_2_^2^(8) loops. The dimers are connected by N—H⋯O hydrogen bonds, forming [011] chains. A C—H⋯O inter­action is also observed.

## Related literature   

For compounds presenting an indole skeleton unit and examples of them, see: Xu *et al.* (2012 ▸); Humphrey & Kuethe (2006 ▸). For methods of synthesis of indoles, see: Jordan *et al.* (2011 ▸); Humphrey & Kuethe (2006 ▸). For the use of Morita–Baylis–Hillman adducts as building blocks for organic synthesis, see: Basavaiah & Veeraraghavaiah (2012 ▸); Coelho *et al.* (2002 ▸).
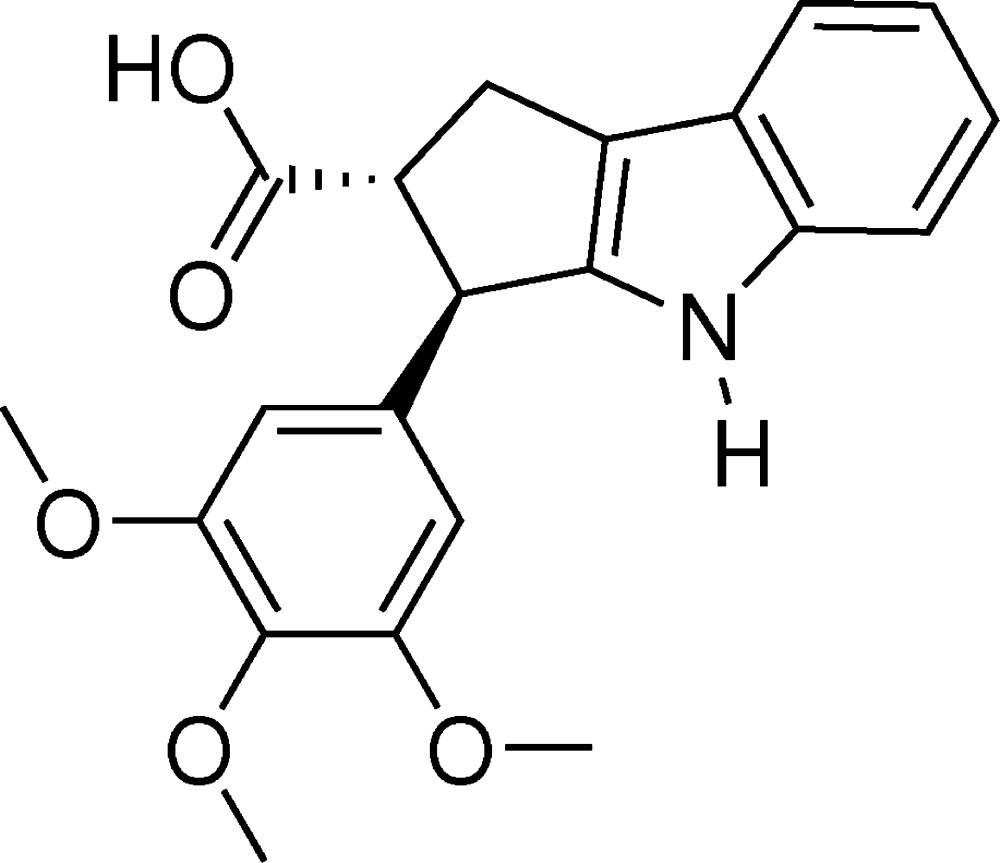


## Experimental   

### Crystal data  

C_21_H_21_NO_5_
*M* *_r_* = 367.39
Triclinic, 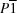

*a* = 7.203 (1) Å
*b* = 9.5844 (12) Å
*c* = 12.9957 (17) Å
α = 91.939 (5)°
β = 97.198 (6)°
γ = 91.716 (5)°
*V* = 889.1 (2) Å^3^
*Z* = 2
Mo *K*α radiation
μ = 0.10 mm^−1^
*T* = 100 K
0.34 × 0.17 × 0.13 mm

### Data collection  

Bruker APEX CCD diffractometer
Absorption correction: multi-scan (*SADABS*; Bruker, 2010 ▸) *T*_min_ = 0.967, *T*_max_ = 0.987
100846 measured reflections
7825 independent reflections
6558 reflections with *I* > 2σ(*I*)
*R*_int_ = 0.031

### Refinement  

*R*[*F* ^2^ > 2σ(*F* ^2^)] = 0.038
*wR*(*F* ^2^) = 0.119
*S* = 0.94
7825 reflections
248 parameters
H-atom parameters constrained
Δρ_max_ = 0.58 e Å^−3^
Δρ_min_ = −0.27 e Å^−3^

### 

Data collection: *APEX2* (Bruker, 2010 ▸); cell refinement: *SAINT* (Bruker, 2010 ▸); data reduction: *SAINT*; program(s) used to solve structure: *SHELXS97* (Sheldrick, 2008 ▸); program(s) used to refine structure: *SHELXLE* (Hübschle *et al.*, 2011 ▸); molecular graphics: *Mercury* (Macrae *et al.*, 2006 ▸); software used to prepare material for publication: *OLEX2* (Dolomanov *et al.*, 2003 ▸) and *publCIF* (Westrip, 2010 ▸).

## Supplementary Material

Crystal structure: contains datablock(s) I, New_Global_Publ_Block. DOI: 10.1107/S2056989015008786/hb7417sup1.cif

Structure factors: contains datablock(s) I. DOI: 10.1107/S2056989015008786/hb7417Isup2.hkl

Supporting information file. DOI: 10.1107/S2056989015008786/hb7417Isup3.cdx

Supporting information file. DOI: 10.1107/S2056989015008786/hb7417Isup4.cml

**Fig. 1.**
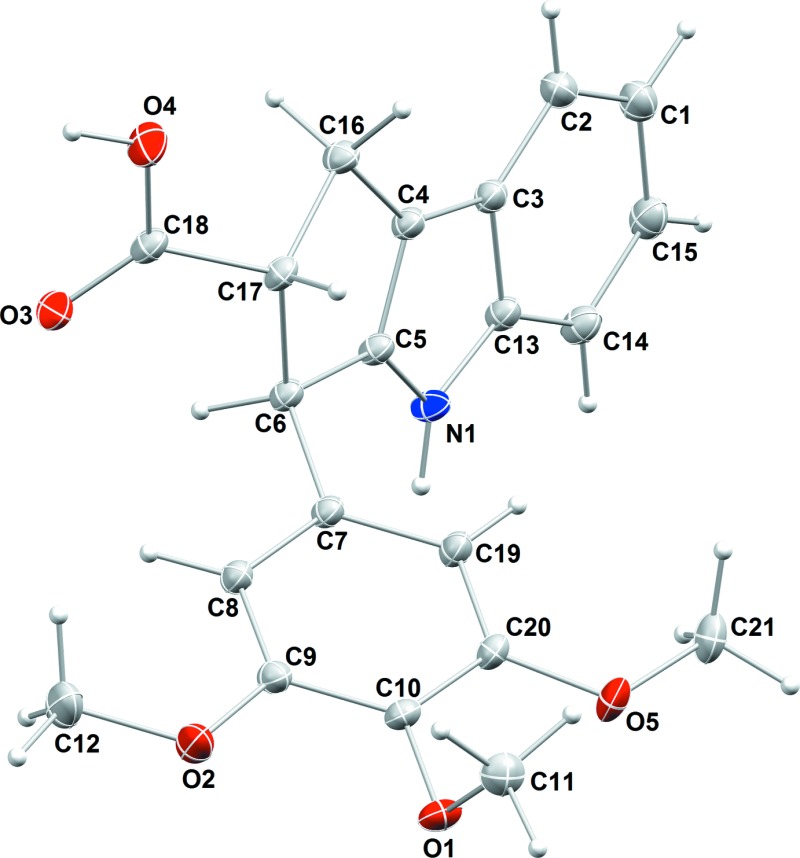
The molecular structure of the title compound with 50% probability displacement ellipsoids.

. DOI: 10.1107/S2056989015008786/hb7417fig1.tif
The mol­ecular structure of the title compound with 50% probability displacement ellipsoids.

**Fig. 2.**
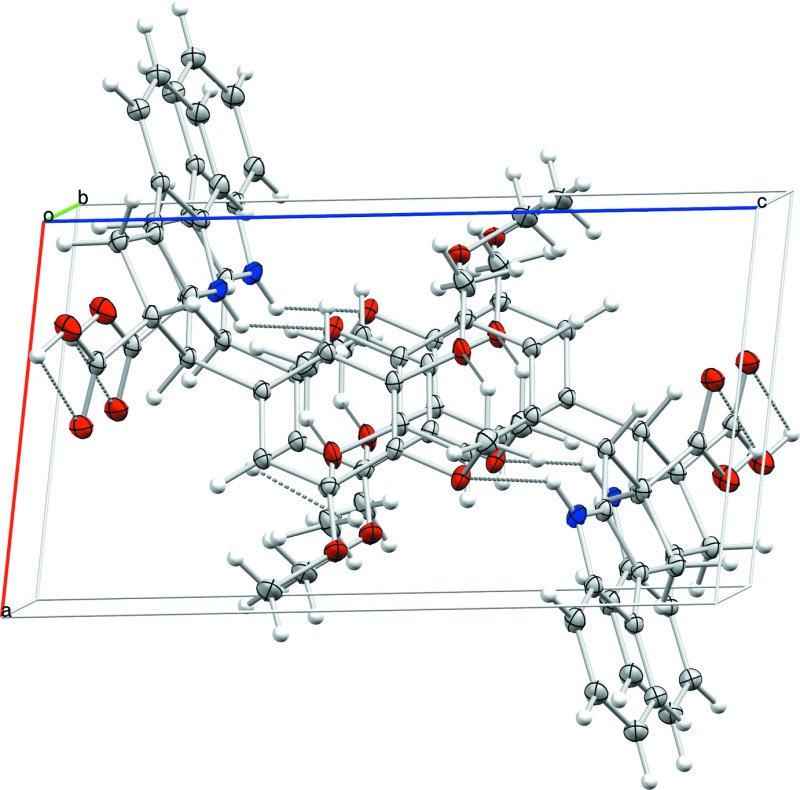
Crystal packing of the title compound, showing hydrogen-bonding interactions.

. DOI: 10.1107/S2056989015008786/hb7417fig2.tif
Crystal packing of the title compound, showing hydrogen-bonding inter­actions.

CCDC reference: 1063387

Additional supporting information:  crystallographic information; 3D view; checkCIF report

## Figures and Tables

**Table 1:** Hydrogen-bond geometry (, )

*D*H*A*	*D*H	H*A*	*D* *A*	*D*H*A*
O4H4O3^i^	0.84	1.84	2.6748(8)	176
N1H1O1^ii^	0.88	2.20	2.9041(8)	136
C12H12*B*O2^iii^	0.98	2.62	3.3905(11)	137

Symmetry codes: (i) 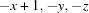; (ii) 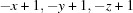; (iii) 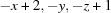.

## supplementary crystallographic information

### S1. Introduction

Indole skeleton is an aromatic heterocycle possessing a benzene ring fused to a pyrrole ring which exhibits a wide range of biological and pharmacological activities. Compounds presenting this moiety have been successfully synthesized and used in medicinal chemistry (Xu *et al.*, 2012). In spite of the number of developed methods for the preparation of indoles (Xu *et al.*, 2012; Humphrey and Kuethe, 2006), our inter­est to use Morita–Baylis–Hillman adducts as building blocks for organic synthesis resulted in a successful stereoselective strategy to obtain compounds of this class.

### S2.1. Synthesis and crystallization

\ The synthesis of 3-(3,4,5-tri­meth­oxy­phenyl)-1,2,3,4-tetra­hydro­cyclo­penta­[b]indole-2-\ carb­oxy­lic acid started with a mixture of 1 mmol of (±)-methyl 2-[hy­droxy­(3,4,5-tri­meth­oxy­phenyl)-methyl]­acrylate (the Morita–Baylis–Hillman adduct), 1.2 mmol of indole and 1 mmol of 2-iodoxybenzoic acid, in aceto­nitrile (5 mL). This mixture was kept under reflux to give 1,3-di­carbonyl compound, which was further reduced by sodium tetra­hydro­borate, resulting in the corresponding β-hy­droxy-carbonyl.

This was then treated with tri­fluoro­methane­sulfonic acid and submitted to basic hydrolysis. The cyclo­penta­[*b*]indole, obtained with excellent diastereoselectivity (>99:1) and overall yield of 70%, was purified by flash chromatography (hexane/ethyl acetate (60:40)). 3-(3,4,5-tri­meth­oxy­phenyl)-1,2,3,4-tetra­hydro­cyclo­penta­[b]indole-2-\ carb­oxy­lic acid was dissolved in 10:1 (v/v) chloro­form/methanol mixture and kept in the freezer to allow slowly formation of irregular colorless single crystals.

### S2.2. Refinement

The positions of hydrogen atoms bound to carbon atoms were idealized and calculated by riding model, with C—H bond lengths of 0.95, 0.98 and 0.99 Å for phenyl, methyl and methyl­ene, respectively. The isotropic displacement parameters values (Uiso(H)) were fixed at 1.5Ueq(C) for methyl H atoms and 1.2Ueq(C) for all other attached H atoms.

### S3. Results and discussion

The molecules of the title compound present a tri­meth­oxy­phenyl ring bonded to a system of three rings, in which an indole skeleton unit is fused to a five-membered ring possessing a carb­oxy­lic unit (Fig. 1). It crystallized in P1 space group and has a conformational structure determined by intra and inter­molecular nonclassical (C—H—O) and inter­molecular (O—H—O and N—H—O) bonding (Table 1, Fig. 2).

All of the rings in the structure are almost planar, with r.m.s. of 0.010, 0.066, 0.006 and 0.006 Å for tri­meth­oxy­phenyl, five-membered, pyrrole and benzene rings, respectively. The three rings fused system is essentially planar (r.m.s. deviation of 0.057 Å) and make a plane-plane angle of 113.35° with the tri­meth­oxy­phenyl ring.

With respect to the pyrrole ring, the benzene ring and the five-membered ring make dihedral angles C14—C13—N1—C5 = 176.67 (7)° and C3—C4—C16—C17 = -175.72 (8)°, respectively. The dihedral angle between the five-membered ring and its tri­meth­oxy­phenyl substituent (C5—C6—C7—C8) is -164.79 (16)°, while that between the five-membered ring and the carb­oxy­lic unit (C16—C17—C18—O4) is -56.30 (8)°, which is consistent with the expected *trans* relative configuration of this isomer.

### Crystal data

**Table:**

C_21_H_21_NO_5_	*Z* = 2
*M**_r_* = 367.39	*F*(000) = 388
Triclinic, *P*1	*D*_x_ = 1.372 Mg m^−^^3^
Hall symbol: -P 1	Mo *K*α radiation, λ = 0.71073 Å
*a* = 7.203 (1) Å	Cell parameters from 9708 reflections
*b* = 9.5844 (12) Å	θ = 2.6–38°
*c* = 12.9957 (17) Å	µ = 0.10 mm^−^^1^
α = 91.939 (5)°	*T* = 100 K
β = 97.198 (6)°	Irregular, colourless
γ = 91.716 (5)°	0.34 × 0.17 × 0.13 mm
*V* = 889.1 (2) Å^3^	

### Data collection

**Table:**

Bruker APEX CCD diffractometer	7825 independent reflections
Radiation source: fine-focus sealed tube	6558 reflections with *I* > 2σ(*I*)
Detector resolution: 8.3333 pixels mm^-1^	*R*_int_ = 0.031
phi and ω scans	θ_max_ = 35.0°, θ_min_ = 1.6°
Absorption correction: multi-scan (*SADABS*; Bruker, 2010)	*h* = −11→11
*T*_min_ = 0.967, *T*_max_ = 0.987	*k* = −15→15
100846 measured reflections	*l* = −20→20

### Refinement

**Table:**

Refinement on *F*^2^	0 restraints
Least-squares matrix: full	Hydrogen site location: inferred from neighbouring sites
*R*[*F*^2^ > 2σ(*F*^2^)] = 0.038	H-atom parameters constrained
*wR*(*F*^2^) = 0.119	*w* = 1/[σ^2^(*F*_o_^2^) + (0.079*P*)^2^ + 0.2107*P*] where *P* = (*F*_o_^2^ + 2*F*_c_^2^)/3
*S* = 0.94	(Δ/σ)_max_ < 0.001
7825 reflections	Δρ_max_ = 0.58 e Å^−^^3^
248 parameters	Δρ_min_ = −0.27 e Å^−^^3^

### Special details

**Table:**

Geometry. All e.s.d.'s (except the e.s.d. in the dihedral angle between two l.s. planes) are estimated using the full covariance matrix. The cell e.s.d.'s are taken into account individually in the estimation of e.s.d.'s in distances, angles and torsion angles; correlations between e.s.d.'s in cell parameters are only used when they are defined by crystal symmetry. An approximate (isotropic) treatment of cell e.s.d.'s is used for estimating e.s.d.'s involving l.s. planes.

### Fractional atomic coordinates and isotropic or equivalent isotropic displacement parameters (Å^2^)

**Table:**

	*x*	*y*	*z*	*U*_iso_*/*U*_eq_	
O1	0.67935 (8)	0.20458 (6)	0.61432 (4)	0.01701 (10)	
O2	0.85593 (8)	0.13066 (7)	0.45301 (4)	0.02026 (11)	
O3	0.52886 (8)	0.14039 (6)	0.07684 (5)	0.02004 (11)	
O4	0.27178 (9)	0.00009 (6)	0.05008 (5)	0.02346 (12)	
H4	0.3380	−0.0455	0.0131	0.035*	
O5	0.36545 (8)	0.34406 (6)	0.59020 (4)	0.01927 (11)	
N1	0.20865 (9)	0.57757 (6)	0.22742 (5)	0.01503 (10)	
H1	0.2993	0.6285	0.2636	0.018*	
C1	−0.33636 (10)	0.65445 (9)	0.10853 (6)	0.01960 (13)	
H1A	−0.4633	0.6671	0.0816	0.024*	
C2	−0.25626 (10)	0.52708 (8)	0.09178 (6)	0.01735 (12)	
H2	−0.3264	0.4538	0.0522	0.021*	
C3	−0.07052 (9)	0.50802 (7)	0.13406 (5)	0.01394 (11)	
C4	0.05337 (9)	0.39331 (7)	0.13915 (5)	0.01372 (11)	
C5	0.21619 (9)	0.43919 (7)	0.19636 (5)	0.01331 (11)	
C6	0.36676 (9)	0.33516 (7)	0.20740 (5)	0.01314 (11)	
H6	0.4670	0.3624	0.1645	0.016*	
C7	0.45411 (9)	0.30909 (7)	0.31710 (5)	0.01289 (11)	
C8	0.62291 (9)	0.24015 (7)	0.33130 (5)	0.01407 (11)	
H8	0.6873	0.2187	0.2736	0.017*	
C9	0.69665 (9)	0.20293 (7)	0.43061 (5)	0.01429 (11)	
C10	0.60455 (9)	0.23835 (7)	0.51579 (5)	0.01402 (11)	
C11	0.58428 (11)	0.08561 (8)	0.65126 (6)	0.02034 (13)	
H11A	0.4493	0.1005	0.6438	0.031*	
H11B	0.6302	0.0737	0.7246	0.031*	
H11C	0.6086	0.0016	0.6106	0.031*	
C12	0.94999 (11)	0.08870 (10)	0.36769 (7)	0.02528 (16)	
H12A	0.8643	0.0311	0.3179	0.038*	
H12B	1.0587	0.0345	0.3927	0.038*	
H12C	0.9918	0.1716	0.3337	0.038*	
C13	0.03194 (10)	0.62141 (7)	0.19116 (5)	0.01434 (11)	
C14	−0.04783 (11)	0.75053 (8)	0.20623 (6)	0.01750 (12)	
H14	0.0223	0.8256	0.2437	0.021*	
C15	−0.23267 (11)	0.76525 (8)	0.16470 (6)	0.01940 (13)	
H15	−0.2905	0.8517	0.1743	0.023*	
C16	0.06472 (10)	0.24706 (7)	0.09663 (5)	0.01538 (12)	
H16A	0.0682	0.2448	0.0207	0.018*	
H16B	−0.0415	0.1870	0.1125	0.018*	
C17	0.25541 (9)	0.20188 (7)	0.15655 (5)	0.01396 (11)	
H17	0.2239	0.1432	0.2146	0.017*	
C18	0.36696 (10)	0.11304 (7)	0.09044 (5)	0.01515 (12)	
C19	0.36205 (9)	0.34551 (7)	0.40179 (5)	0.01444 (11)	
H19	0.2477	0.3931	0.3918	0.017*	
C20	0.43904 (10)	0.31162 (7)	0.50143 (5)	0.01433 (11)	
C21	0.19448 (12)	0.41674 (9)	0.58029 (6)	0.02137 (14)	
H21A	0.2090	0.5011	0.5411	0.032*	
H21B	0.1631	0.4429	0.6494	0.032*	
H21C	0.0939	0.3561	0.5435	0.032*	

### Atomic displacement parameters (Å^2^)

**Table:**

	*U*^11^	*U*^22^	*U*^33^	*U*^12^	*U*^13^	*U*^23^
O1	0.0210 (2)	0.0173 (2)	0.0115 (2)	−0.00177 (18)	−0.00212 (16)	0.00000 (16)
O2	0.0164 (2)	0.0267 (3)	0.0175 (2)	0.00815 (19)	0.00017 (17)	−0.00044 (19)
O3	0.0183 (2)	0.0206 (3)	0.0210 (2)	0.00222 (19)	0.00369 (18)	−0.00790 (19)
O4	0.0243 (3)	0.0181 (3)	0.0283 (3)	−0.0019 (2)	0.0082 (2)	−0.0119 (2)
O5	0.0259 (3)	0.0208 (3)	0.0124 (2)	0.0063 (2)	0.00682 (18)	−0.00088 (18)
N1	0.0170 (2)	0.0125 (2)	0.0146 (2)	0.00230 (18)	−0.00148 (18)	−0.00317 (18)
C1	0.0167 (3)	0.0230 (3)	0.0192 (3)	0.0042 (2)	0.0019 (2)	0.0012 (2)
C2	0.0155 (3)	0.0197 (3)	0.0166 (3)	0.0007 (2)	0.0015 (2)	0.0002 (2)
C3	0.0151 (3)	0.0150 (3)	0.0116 (2)	0.0016 (2)	0.00173 (19)	−0.0009 (2)
C4	0.0155 (3)	0.0137 (3)	0.0118 (2)	0.0010 (2)	0.00152 (19)	−0.00208 (19)
C5	0.0160 (3)	0.0121 (3)	0.0115 (2)	0.0017 (2)	0.00085 (19)	−0.00173 (19)
C6	0.0153 (2)	0.0127 (3)	0.0113 (2)	0.0017 (2)	0.00156 (19)	−0.00173 (19)
C7	0.0147 (2)	0.0127 (3)	0.0111 (2)	0.0013 (2)	0.00167 (19)	−0.00187 (19)
C8	0.0146 (2)	0.0153 (3)	0.0123 (2)	0.0017 (2)	0.00190 (19)	−0.0019 (2)
C9	0.0136 (2)	0.0149 (3)	0.0139 (2)	0.0015 (2)	0.00027 (19)	−0.0019 (2)
C10	0.0163 (3)	0.0142 (3)	0.0109 (2)	−0.0002 (2)	−0.00008 (19)	−0.00137 (19)
C11	0.0230 (3)	0.0187 (3)	0.0195 (3)	0.0007 (2)	0.0022 (2)	0.0047 (2)
C12	0.0191 (3)	0.0344 (4)	0.0229 (3)	0.0105 (3)	0.0035 (3)	−0.0029 (3)
C13	0.0168 (3)	0.0141 (3)	0.0120 (2)	0.0029 (2)	0.00133 (19)	−0.00108 (19)
C14	0.0212 (3)	0.0152 (3)	0.0158 (3)	0.0046 (2)	0.0008 (2)	−0.0018 (2)
C15	0.0206 (3)	0.0199 (3)	0.0181 (3)	0.0071 (2)	0.0024 (2)	0.0002 (2)
C16	0.0169 (3)	0.0139 (3)	0.0148 (3)	0.0006 (2)	0.0008 (2)	−0.0035 (2)
C17	0.0176 (3)	0.0124 (3)	0.0119 (2)	0.0016 (2)	0.00258 (19)	−0.00245 (19)
C18	0.0195 (3)	0.0134 (3)	0.0124 (2)	0.0028 (2)	0.0016 (2)	−0.0023 (2)
C19	0.0163 (3)	0.0156 (3)	0.0117 (2)	0.0032 (2)	0.0025 (2)	−0.0011 (2)
C20	0.0179 (3)	0.0139 (3)	0.0115 (2)	0.0014 (2)	0.0033 (2)	−0.00188 (19)
C21	0.0257 (3)	0.0198 (3)	0.0208 (3)	0.0062 (3)	0.0110 (3)	−0.0001 (2)

### Geometric parameters (Å, º)

**Table:**

O1—C10	1.3785 (8)	C7—C8	1.3961 (9)
O1—C11	1.4396 (10)	C8—C9	1.3949 (10)
O2—C9	1.3632 (9)	C8—H8	0.9500
O2—C12	1.4229 (10)	C9—C10	1.3974 (9)
O3—C18	1.2232 (9)	C10—C20	1.3973 (10)
O4—C18	1.3206 (9)	C11—H11A	0.9800
O4—H4	0.8400	C11—H11B	0.9800
O5—C20	1.3591 (8)	C11—H11C	0.9800
O5—C21	1.4269 (10)	C12—H12A	0.9800
N1—C5	1.3779 (9)	C12—H12B	0.9800
N1—C13	1.3834 (9)	C12—H12C	0.9800
N1—H1	0.8800	C13—C14	1.3980 (10)
C1—C2	1.3879 (11)	C14—C15	1.3857 (11)
C1—C15	1.4078 (11)	C14—H14	0.9500
C1—H1A	0.9500	C15—H15	0.9500
C2—C3	1.4009 (10)	C16—C17	1.5719 (10)
C2—H2	0.9500	C16—H16A	0.9900
C3—C13	1.4271 (10)	C16—H16B	0.9900
C3—C4	1.4347 (9)	C17—C18	1.5078 (9)
C4—C5	1.3601 (9)	C17—H17	1.0000
C4—C16	1.4975 (10)	C19—C20	1.3961 (9)
C5—C6	1.4921 (9)	C19—H19	0.9500
C6—C7	1.5164 (9)	C21—H21A	0.9800
C6—C17	1.5686 (10)	C21—H21B	0.9800
C6—H6	1.0000	C21—H21C	0.9800
C7—C19	1.3942 (9)		
			
C10—O1—C11	112.48 (6)	H11A—C11—H11C	109.5
C9—O2—C12	116.77 (6)	H11B—C11—H11C	109.5
C18—O4—H4	109.5	O2—C12—H12A	109.5
C20—O5—C21	117.21 (6)	O2—C12—H12B	109.5
C5—N1—C13	107.21 (6)	H12A—C12—H12B	109.5
C5—N1—H1	126.4	O2—C12—H12C	109.5
C13—N1—H1	126.4	H12A—C12—H12C	109.5
C2—C1—C15	121.12 (7)	H12B—C12—H12C	109.5
C2—C1—H1A	119.4	N1—C13—C14	129.41 (7)
C15—C1—H1A	119.4	N1—C13—C3	108.66 (6)
C1—C2—C3	119.10 (7)	C14—C13—C3	121.91 (6)
C1—C2—H2	120.5	C15—C14—C13	117.73 (7)
C3—C2—H2	120.5	C15—C14—H14	121.1
C2—C3—C13	118.90 (6)	C13—C14—H14	121.1
C2—C3—C4	135.29 (7)	C14—C15—C1	121.22 (7)
C13—C3—C4	105.75 (6)	C14—C15—H15	119.4
C5—C4—C3	107.07 (6)	C1—C15—H15	119.4
C5—C4—C16	112.09 (6)	C4—C16—C17	101.36 (5)
C3—C4—C16	140.65 (6)	C4—C16—H16A	111.5
C4—C5—N1	111.28 (6)	C17—C16—H16A	111.5
C4—C5—C6	115.13 (6)	C4—C16—H16B	111.5
N1—C5—C6	133.48 (6)	C17—C16—H16B	111.5
C5—C6—C7	116.44 (5)	H16A—C16—H16B	109.3
C5—C6—C17	100.20 (5)	C18—C17—C6	113.56 (6)
C7—C6—C17	111.15 (5)	C18—C17—C16	113.05 (5)
C5—C6—H6	109.5	C6—C17—C16	109.05 (5)
C7—C6—H6	109.5	C18—C17—H17	106.9
C17—C6—H6	109.5	C6—C17—H17	106.9
C19—C7—C8	120.56 (6)	C16—C17—H17	106.9
C19—C7—C6	120.63 (6)	O3—C18—O4	123.34 (6)
C8—C7—C6	118.70 (6)	O3—C18—C17	124.14 (6)
C9—C8—C7	119.72 (6)	O4—C18—C17	112.51 (6)
C9—C8—H8	120.1	C7—C19—C20	119.54 (6)
C7—C8—H8	120.1	C7—C19—H19	120.2
O2—C9—C8	124.76 (6)	C20—C19—H19	120.2
O2—C9—C10	115.23 (6)	O5—C20—C19	125.19 (6)
C8—C9—C10	120.01 (6)	O5—C20—C10	114.64 (6)
O1—C10—C20	119.89 (6)	C19—C20—C10	120.17 (6)
O1—C10—C9	120.14 (6)	O5—C21—H21A	109.5
C20—C10—C9	119.94 (6)	O5—C21—H21B	109.5
O1—C11—H11A	109.5	H21A—C21—H21B	109.5
O1—C11—H11B	109.5	O5—C21—H21C	109.5
H11A—C11—H11B	109.5	H21A—C21—H21C	109.5
O1—C11—H11C	109.5	H21B—C21—H21C	109.5

### Hydrogen-bond geometry (Å, º)

**Table:**

*D*—H···*A*	*D*—H	H···*A*	*D*···*A*	*D*—H···*A*
O4—H4···O3^i^	0.84	1.84	2.6748 (8)	176
N1—H1···O1^ii^	0.88	2.20	2.9041 (8)	136
C12—H12*B*···O2^iii^	0.98	2.62	3.3905 (11)	137

Symmetry codes: (i) −*x*+1, −*y*, −*z*; (ii) −*x*+1, −*y*+1, −*z*+1; (iii) −*x*+2, −*y*, −*z*+1.
