# Supplementary figures and images for: Crystal structure of 3-(3,4,5-tri­meth­oxy­phen­yl)-1,2,3,4-tetra­hydro­cyclo­penta[b]indole-2-carb­oxy­lic acid
Source: Acta Crystallogr E Crystallogr Commun. 2015 May 13;71(Pt 6):o395–6. doi: 10.1107/S2056989015008786 (PMC4459319; doi:10.1107/S2056989015008786)

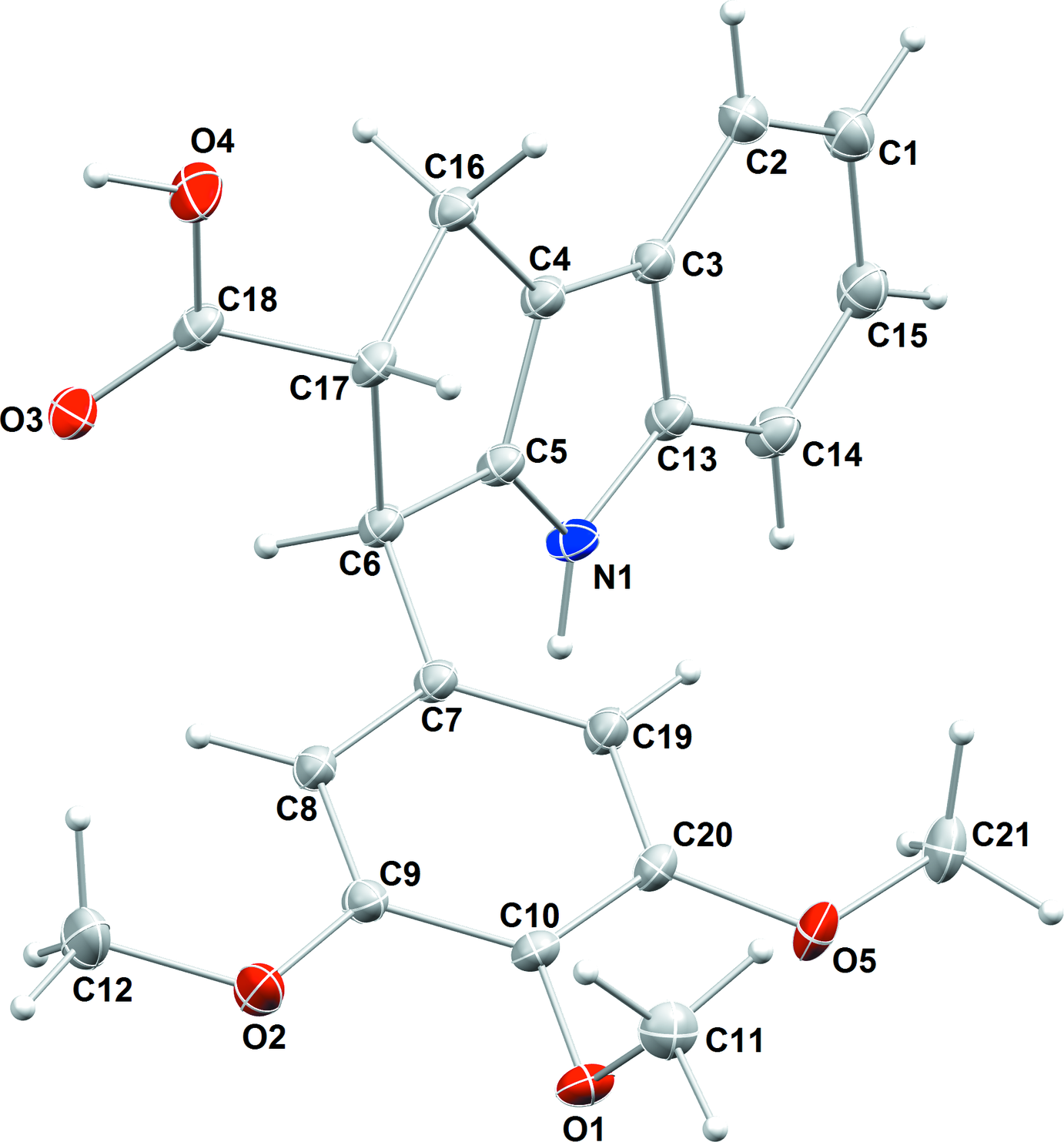

Supplement: Supplementary file 5 [file e-71-0o395-fig1.tif]

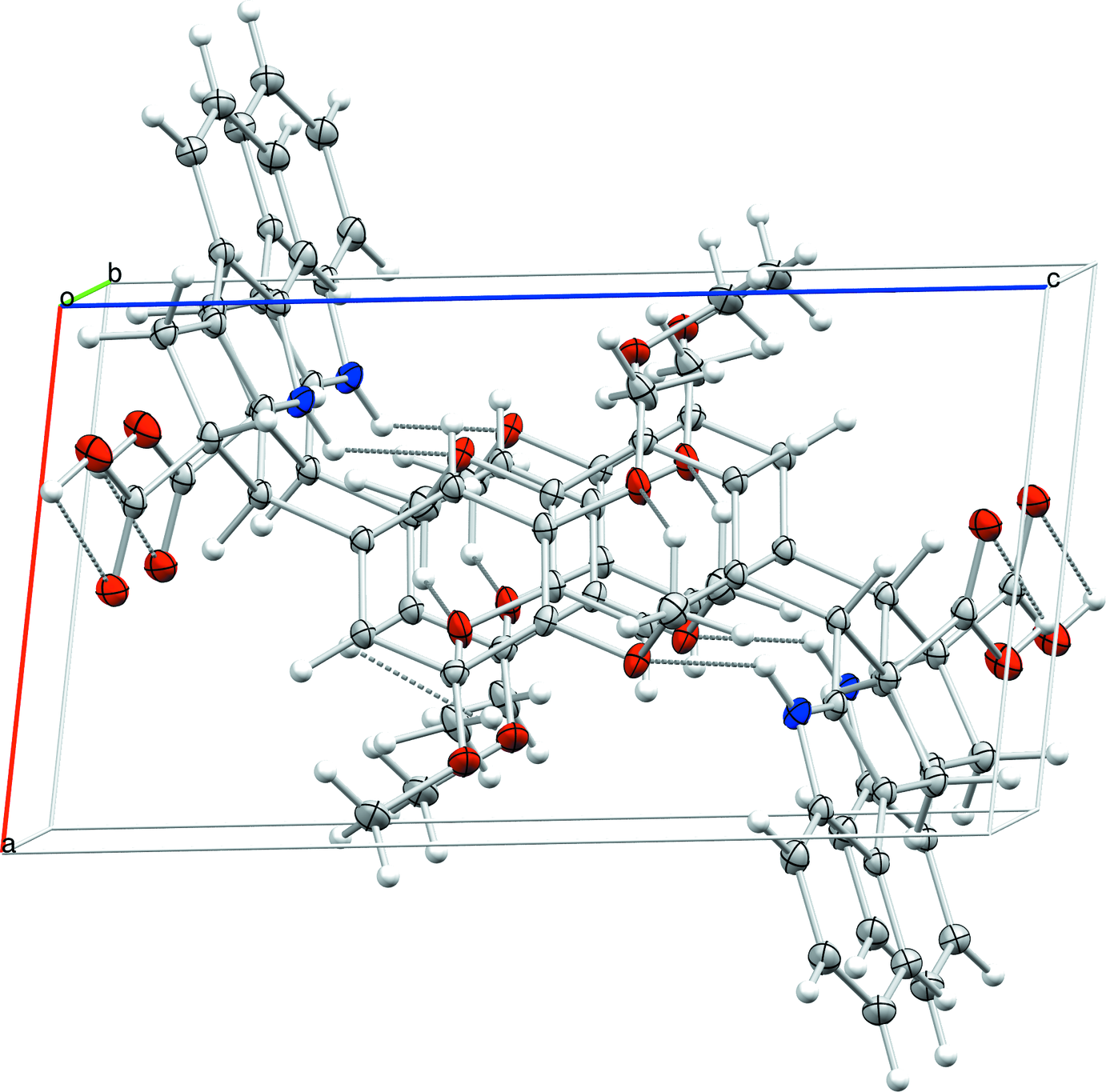

Supplement: Supplementary file 6 [file e-71-0o395-fig2.tif]
